# Supplementary material for: Epstein-Barr virus ensures B cell survival by uniquely modulating apoptosis at early and late times after infection
Source: eLife. 2017 Apr 20;6:e22509. doi: 10.7554/eLife.22509 (PMC5425254; doi:10.7554/eLife.22509)
Supplement: Supplementary file 1. — DOI: http://dx.doi.org/10.7554/eLife.22509.022 [file elife-22509-supp1.docx]

**Antibodies Used**

Antibodies used for western blot and chromatin immunoprecipitation are included below:

| Antibodies Used for Western Blot: | | |
| --- | --- | --- |
| BCL-2 | BD Biosciences, BD551107 | RRID:AB_394048 |
| MCL-1 | Santa Cruz, sc-819 | RRID:AB_2144105 |
| MAGOH | Santa Cruz, sc-56724 | RRID:AB_629914 |
| EBNA3A | Exalpha, F115P | (no RRID) |
| EBNA3C | Exalpha, F125P | (no RRID) |
| VDAC | EMD Millipore, AB10527 | RRID:AB_10806766 |
| β-ACTIN | Rockland, 600-401-886 | RRID:AB_2612818 |
| GAPDH | BioChain Institute, #Y3322 | (no RRID) |
| Antibodies Used for ChIP: | | |
| Total RNA pol II | Santa Cruz, sc-899 | RRID:AB_632359 |
| RNA pol II CTD repeat YSPTSPS (phospho S5) | Abcam, ab5131 | RRID:AB_449369 |
| H3K27ac | Millipore, 17-683 | RRID:AB_1977529 |
| H3K9ac | Millipore 17-658 | RRID:AB_1587124 |
| H3K4me3 | Millipore, 17-614 | RRID:AB_1587135 |

**Oligos Used**

Oligos used for qPCR, ChIP-qPCR, and Chromatin Conformation Capture are included below:

| Oligos used for qPCR (5’ 🡺 3’): | | |
| --- | --- | --- |
| Oligo Name | Forward Primer | Reverse Primer |
| MCL-1 | GTGCCTTTGTGGCTAAACACT | AGTCCCGTTTTGTCCTTACGA |
| BCL-2 | GGTGGGGTCATGTGTGTGG | CGGTTCAGGTACTCAGTCATCC |
| BFL-1 | TTACAGGCTGGCTCAGGACT | AGCACTCTGGACGTTTTGCT |
| W Promoter (Wp) | CGCCAGGAGTCCACACAAAT | GAGGGGACCCTCTGGCC |
| C Promoter (Cp) | AATCATCTAAACCGACTGAAGAAACAG | GAGGGGACCCTCTGGCC |
| Latent W2-BHRF1 | TGGTAAGCGGTTCACCTTCAG | TCCCGTATACACAGGGCTAACAGT |
| EBNA2 | GCTTAGCCAGTAACCCAGCACT | TGCTTAGAAGGTTGTTGGCATG |
| EBNA3A | CTGCAGCCCAGAGAGTAGTC | GCCTGTCCTTGTCCATTTTG |
| EBNA3C | CTGCAGCCCAGAGAGTAGTC | TCCATGGTGGGTCTTAAAGG |
| SETDB1 | TCCATGGCATGCTGGAGCGG | GAGAGGGTTCTTGCCCCGGT |
| Oligos used for ChIP (5’ 🡺 3’): | | |
| Oligo Name | Forward Primer | Reverse Primer |
| Myo | GGAGAAAGAAGGGGAATCACA | GATAAATATAGCCAACGCCACA |
| CXCL10 TSS | TCCCTCCCTAATTCTGATTGG | AGCAGAGGGAAATTCCGTAAC |
| miR221/222 TSS | TCCAGCACCTAAGAAAATATGTGGC | CCCATGTACGTAATTTTAAACAACCTC |
| BFL-1_1 | GCAATGGGCACACCTGAAAG | GCCAAAGGGTAAAGCTCAAC |
| BFL-1_2 | ACAGTGGTTACCTCTTGGGAGA | CCTGTGTTGAAACTCATGTTGGTA |
| BFL-1_TSS | TGGACCTGATCCAGGTTGTGGTA | TGCTCTCCACCAGGCAGAAG |
| BFL-1_3 | AGGAATTTGGCCTCCCAATCA | TTTCTCCAGCGACCATGAGTT |
| BFL-1_4 | AATCATAACTGCATGTGCCAAGTGC | CCTACACATGCCAAAGCTTCAGC |
| Oligos used for CCC (5’ 🡺 3’): | | |
| BFL-1 HindIII Fragment | Forward Primer | Modifications |
| TSS Probe | TATAACCTGGGAGTTGAAGGGTTTTGCGGT | (5’FAM, 3’TAMRA) |
| TSS Constant | TTAGAAACAGCACTTCCCTTTTTACTG |  |
| +2 | GTGTAAGACTTCCAGTTGCTCATT |  |
| +1 | GAAAGCATACAGGATCCCTGCC |  |
| -1 | CAACTACCCACCTTACCTCTCTCC |  |
| -3 | CCGGATGAGATTACCATGGAGC |  |
| -8 | GACTGGGAGAATTAGGGAGCTC |  |
| -12 | CCCTTACCTTGTTTCTGAGCTGAG |  |
| -14 | CACAGAACTGTGCAGCCAAG |  |

| Short-guide RNA sequences: | | |
| --- | --- | --- |
| Name | Sequence (5’🡺3) PAM site not included | Location |
| BFL1 | CTTGGACCTGATCCAGGTTG | BCL2A1 Exon 1 |
| AAV | GGGGCCACTAGGGACAGGAT | AAVS1 |

| Primers for generating EBNA3-STOP Mutants: (from Reza Djavadian) | |
| --- | --- |
| Name | Sequence (5’🡺3) PAM site not included |
| EBNA3A-STOP Primer 1 | GGTGTTGGTGAGTCACACTTTTGTTGCAGACAAAATGGACTAGGATAGGCCGGGTCCCCCGGCCCTAGGATGACGACGATAAGTAGGG |
| EBNA3A-STOP Primer 2 | CTTCTTCCATGTTGTCATCCAGGGCCGGGGGACCCGGCCTATCCTAGTCCATTTTGTCTGCAACAACAACCAATTAACCAATTCTGATTAG |
| EBNA3C-STOP Primer 1 | AGATGAGGTAGAAATTTTGCATATTTTCAGACCCACCATGTAATCATTTGAAGGACAGGGGAGGATGACGACGATAAGTAGGG |
| EBNA3C-STOP Primer 2 | TCGGGTGACTGTCTAGAGTCCCCCTGTCCTTCAAATGATTACATGGTGGGTCTGAAAATATCAACCAATTAACCAATTCTGATTAG |
| BAC-KAN-F | CGGGCGTATTTTTTGAGTTATCGAGATTTTCAGGAGCTAAGGAAGCTAAAATGAGCCATATTCAACGGGAAAC |
| BAC-KAN-R | CAGGCGTAGCAACCAGGCGTTTAAGGGCACCAATAACTGCCTTAAAAAAATTAGAAAAACTCATCGAGCATC |
